# A Rare Case of *Cladophialophora bantiana* Intracranial Infection: Highlighting the Utility of Next-Generation Sequencing in Diagnosis

**DOI:** 10.1155/2024/8892177

**Published:** 2024-09-28

**Authors:** Melissa Whitman, Nicole Vissichelli

**Affiliations:** Division of Infectious Diseases Virginia Commonwealth University Health System, Richmond, Virginia 23298-0049, USA

**Keywords:** brain abscess, cerebral phaeohyphomycosis, *Cladophialophora bantiana*, dematiaceous fungi

## Abstract

*Cladophialophora bantiana* is a dematiaceous fungi and the most common cause of cerebral phaeohyphomycosis. Here, we report a rare case of cerebral ventriculitis with development of a cerebral abscess due to *C. bantiana* diagnosed by cell-free DNA next-generation sequencing. Noninvasive diagnostics led to earlier disease identification and initiation of antifungal therapy, which has the potential to reduce mortality in this highly fatal disease.

## 1. Introduction

*Cladophialophora bantiana* is a soil saprophyte found globally [1]. It belongs to the dematiaceous mold class, characterized by its dark color due to presence of melanin in the cell wall [1]. *C. bantiana* is thought to colonize the pulmonary system through inhalation, and then translocate to the central nervous system (CNS) via hematogenous spread [2]. The primary clinical manifestation of *C. bantiana* infection is brain abscess. Diagnosis is challenging and typically involves aspiration or excision of the brain abscess for fungal culture growth. The mean time to diagnosis from symptom onset has been estimated at 115 days [3]. Suspected factors contributing to this delay include misdiagnosis, disease rarity, need for neurosurgical intervention, and the prolonged time needed for proper organism identification on culture. Symptoms of *C. bantiana* intracranial abscess are often nonspecific, primarily including headache, and less commonly fever, seizures, and focal neurologic deficits [4, 5]. CNS radiographic findings can encompass solitary abscess, multiple brain abscesses, or poorly demarcated cerebritis [4].

Since its initial identification in the 1940s, mortality rate has remained elevated at around 70%, regardless of immune status [1, 5]. Given this high mortality, early diagnosis is essential for any hope for clinical cure. To date, the only intervention associated with improved survival is complete surgical excision [2]. Combination antifungal regimens are often employed in treatment, with some retrospective reviews suggesting improved survival over monotherapy [1]. It is essential to identify CNS infections caused by *C. bantiana* to attempt complete surgical excision and to initiate combination antifungals with adequate CNS concentrations, as these are the only two interventions shown to improve outcomes [1, 2]. Given the delays in diagnosis using traditional culture, next-generation sequencing and molecular diagnostics have shown promise to facilitate earlier diagnosis and therefore improved outcomes. Here, we describe a case of *C. bantiana* ventriculitis and cerebritis diagnosed by plasma microbial cell-free DNA next-generation sequencing (mcfDNA-NGS).

## 2. Case Presentation

A 56-year-old immunocompromised male with a past medical history of renal transplant and failed pancreas transplant 5 years prior presented to an outside hospital with 4 months of intermittent confusion and headaches, with worsening of symptoms over the past week. Details of the patient's initial transplant including his induction regimen were not available for review. Maintenance immunosuppressive medications since transplant included mycophenolate and tacrolimus. He had no evidence of kidney transplant rejection and no recent changes in his immunosuppressive regimen in the months prior to presentation. Two months prior to onset of symptoms, he was hospitalized for fevers and confusion, with no acute intracranial findings seen on noncontrasted computed tomography (CT) imaging. Patient and family declined lumbar puncture, and his encephalopathy resolved prior to date of hospital discharge. Two months later (Day 0), he presented again to an outside hospital with fevers and confusion. Noncontrasted CT imaging on Day 0 noted new subcortical and periventricular hypodensities, most prominent along the right lateral ventricle. A lumbar puncture was obtained and revealed significant pleocytosis with 2,008 nucleated cells/mm^3^ with neutrophil predominance, elevated protein to 451 mg/dL, but no organism was identified on culture nor on a meningitis/encephalitis panel by multiplex polymerase chain reaction (PCR) (ARUP laboratories, Salt Lake City, UT). His mental status continued to decline, and on Day 5, he developed acute weakness in his right lower extremity. He was transferred to our center for further evaluation given his renal transplant history.

**Figure 1:**
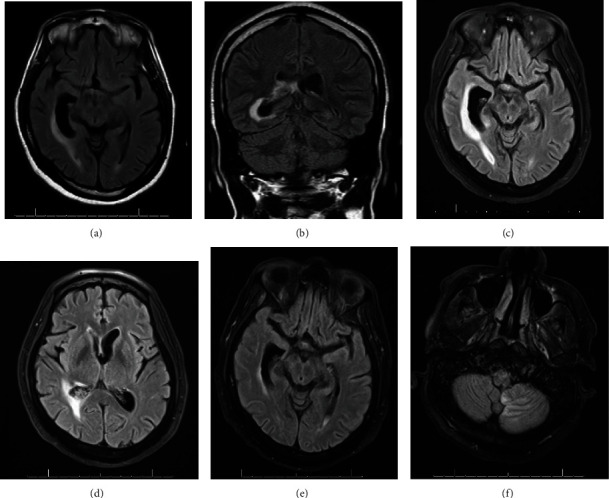
(a, b) (MRI brain T2/FLAIR): panels (a) [axial] and (b) [coronal] with hyperintense signal in the periventricular white matter along the right occipital horn with asymmetric dilation of the temporal horn of the right lateral ventricle. (c, d) (MRI brain T2 qTSE [axial]): panel (c) is showing asymmetric enhancement of the choroid plexus within the right lateral ventricle, mild ependymal enhancement, and foci of high diffusion signal within the lateral ventricles bilaterally, which are compatible with ventriculitis. Panel (d) additionally with new high diffusion signal along the occipital horn of the left lateral ventricle and new acute ischemic infarction of the lower cervicomedullary junction (not shown). (e, f) (MRI T2/FLAIR [axial]): panel (e) shows the right ventricle asymmetrically enlarged relative to the left with ongoing but decreased adjacent hyperintensity. Focal diffusion restriction within the left lateral ventricle was more prominent and was thought to represent evolving abscess formation of the choroid plexus in the setting of ventriculitis, and layering pus visualized in the posterior horn left lateral ventricle. Panel (f) shows a new area of diffusion restriction within the left inferior vermis and left inferior cerebellar hemisphere with associated cytotoxic edema concerning for acute/subacute infarct.

At the time of presentation to our center (Day 7), pertinent physical exam findings included one-out-of-five right lower extremity weakness with diminished sensation to both lower extremities. He had no rash or evidence of cutaneous infection. Heart and lung examination were within normal limits. He had no pertinent travel history, risk factors for tuberculosis or tuberculosis exposures, animal exposures, or sick contacts. The wife did report that the patient spent significant time outdoors including near a construction site very close to their home. Laboratory results revealed leukocytosis to 15,000 cells/*μ*L, creatinine of 1.47 mg/dL (around his baseline) normal transaminases, and a low tacrolimus level to 6.8 ng/mL. A lumbar puncture was pursued and revealed a normal opening pressure, 2,664 nucleated cells/mm^3^ (87% neutrophils), glucose 18 mg/dL (normal 50–75 mg/dL), and elevated protein to 175 mg/dL (normal 15–40 mg/dL). Other pertinent results included negative serum beta-D-glucan, serum cryptococcal antigen, HIV antibody/antigen, and syphilis antibody. Cerebral spinal fluid (CSF) bacterial, fungal, and acid-fast bacilli (AFB) cultures were negative. A CSF sample was sent for next-generation 16S sequencing (Molecular Diagnostics, University of Washington, Renton, WA), but no organism was identified at a significant level. Notably, a beta-D-glucan testing sent from CSF was elevated to 273 pg/mL (normal less than 30 pg/mL). Magnetic resonance imaging (MRI) of the brain showed a new ischemic infarction of the lower medulla as well as ventriculitis and ependymitis of the right choroid plexus temporal horn with partial ventricular entrapment (Figures 1(a) and 1(b)).

Plasma mcfDNA-NGS (Karius Test, Karius, Redwood City, CA) resulted with significant detection of *C. bantiana* at 11 DNA molecules/mL (Day 9). Treatment was initiated with intravenous liposomal amphotericin-B (L-Amb) 7 mg/kg per day, flucytosine 100 mg/kg/day in four divided doses, and voriconazole 3 mg/kg every 12 h with a target trough level between 1.5 and 5 mcg/mL. The neurosurgical service was consulted for potential surgical intervention; however, due to the location of the lesions and lack of discrete abscess formation, the risks of surgical intervention were felt to outweigh the benefits. Repeat MRI of the brain on Day 15 of hospitalization (Figures 1(c) and 1(d)) showed stable enhancement of the right choroid plexus of the temporal horn without worsening hydrocephalus or ventricular entrapment. While on L-Amb, renal function was slightly elevated from baseline with a serum creatine (Cr) of 1.5–1.7 mg/dL (baseline creatinine of 1.3). However, no acute kidney injury or severe electrolyte derangements developed. L-Amb was discontinued on hospital Day 21 after the patient demonstrated clinical stability and therapeutic voriconazole levels. Renal function remained stable with Cr of 1.64 mg/dL at time of L-Amb discontinuation. Intravenous voriconazole was transitioned to oral once the trough level was therapeutic and flucytosine was continued, with a plan for at least 12 months of therapy and repeat MRI of the brain in 4 weeks.

On Day 26, the patient developed sudden onset right-sided weakness. A repeat MRI of the brain demonstrated a new left cerebellar ischemic stroke as well as new findings of possible abscess in the posterior horn of the left lateral ventricle (Figures 1(e) and 1(f)) and L-Amb was resumed. His hospital course was additionally complicated by norovirus infection, worsening leukocytosis, and hemodynamic instability. While his colitis and leukocytosis improved, his mental status continued to decline during his hospitalization. After further discussion with the family, the patient was transitioned to comfort care and was discharged to home with hospice services on hospital Day 29. Ultimately, the patient passed 11 days after hospital discharge.

## 3. Discussion

Intracranial infections caused by *C. bantiana* have a high mortality rate of 65%–70% [3, 4]. Treatment guidance for this intracranial infection is minimal and limited to retrospective case series. Based on current guidance, the management of *C. bantiana* infections includes combination antifungal therapy along with complete surgical excision of the intracranial abscess [1, 3, 4]. Newer generation triazoles such as voriconazole are thought to have better CNS penetration and activity against *C. bantiana* [6, 7]. The combination of second-generation antifungals as well as advancements in surgical techniques hold promise for improved outcomes, yet without enhanced diagnostic methods, high mortality rates for this infection will likely persist.

A delay in diagnosis is a significant barrier to timely intervention and improved outcomes. Diagnosis of *C. bantiana* and other neurotropic fungi requires culture of infected intracranial tissue or pus aspirated from the formed abscess [8]. CSF fungal culture in our patient was negative, which was expected due to the localized nature of intracranial infection and lack of meningeal involvement. In a pooled analysis of 120 *C. bantiana* cases, only 13.3% presented as ventriculitis and only 18 of the 120 cases demonstrated meningeal involvement [8]. In our patient with ventriculitis and no discrete intracranial abscess, surgical debridement for culture or brain biopsy was not possible.

Due to challenges in diagnosing these atypical mold infections, the 2014 European Society of Clinical Microbiology and Infectious Diseases (ESCMID) guidelines for the management of systemic phaeohyphomycosis recommend both culture and sequencing of the internal transcribed spacer (ITS) region of its rDNA [9]. There is additional growing literature that supports the utility of plasma next-generation sequencing techniques in difficult-to-diagnose fungal and mold infections, particularly in immunocompromised patients. Plasma cell-free DNA next-generation sequencing (cfDNA-NGS) was used to identify *C. bantiana* prior to culture positivity in a liver transplant recipient with multiple brain abscesses, which was reported in 2022 [10]. This led to an earlier initiation of antifungals and surgical debridement. In another case report, an 18-year-old man with acute lymphoblastic leukemia was diagnosed with disseminated *Rhizomucor pusillus* infection after the organism was identified by cfDNA-NGS testing [11]. Due to antifungal exposure and delays in obtaining a biopsy, this result led to much earlier targeted intervention compared to awaiting a diagnosis by culture or histopathology. In a 2023 case series, plasma from 35 hematopoietic stem cell transplant recipients with confirmed invasive mold infections was retrospectively analyzed and mcfDNA-NGS was able to identify pathogenic molds up to 3 weeks prior to culture diagnosis [12].

Interestingly, next-generation sequencing of CSF for bacterial, fungal, and AFB pathogens was not diagnostic in our case, likely due to the low concentration of organism in the meninges. We suspect that direct sequencing from brain tissue rather than from CSF may have been diagnostic, but this was not performed due to procedural risk. Metagenomic next-generation sequencing has shown promising results, with a pooled meta-analysis demonstrating 96% specificity for diagnosis of CSF infection [13]. However, results have not been consistent, and sensitivity remains lower than expected, at around 77% [13].

Our case is one of the first to describe intracranial *C bantiana* ventriculitis that was diagnosed through plasma cfDNA-NGS. A similar published case report described a 9-year-old patient with acute-onset headache, ophthalmoplegia, and ataxia with numerous negative fungal CSF cultures spanning over multiple months [14]. Plasma cfDNA-NGS testing was sent and led to the diagnosis of intracranial *C. bantiana* infection. As noninvasive molecular sequencing techniques become more widely accessible, we suspect more published cases will describe the utility of these tests for earlier diagnosis and intervention in mold infections. This case underscores the role of advanced molecular diagnostics in achieving a diagnosis that would have otherwise required high-risk invasive procedures.

## Data Availability

Data sharing is not applicable to this article as no new data were created or analyzed in this study.
